# Supplementary material for: Identification of two-dimensional layered dielectrics from first principles
Source: Nat Commun. 2021 Aug 19;12:5051. doi: 10.1038/s41467-021-25310-2 (PMC8376903; doi:10.1038/s41467-021-25310-2)
Supplement: Supplementary file 1 — Supplementary Information [file 41467_2021_25310_MOESM1_ESM.pdf]

Supplementary Information: Identification of Two-Dimensional Layered Dielectrics from First Principles

Mehrdad Rostami Osanloo[1], Maarten L. Van de Put[2], Ali Saadat[2], and William G. Vandenberghe[2]

[1] Department of Physics, The University of Texas at Dallas, Richardson, TX 75080, USA
[2] Department of Materials Science and Engineering, The University of Texas at Dallas, Richardson, TX 75080, USA
Corresponding Author: William. G. Vandenberghe (william.vandenberghe@utdallas.edu)

Supplementary Table 1. Four major categories of 2D layered materials based on their chemical formula, space group (number) and crystal systems. Category 1 consists of three subcategories as appeared in a, b and c.

<table>
<tr><th></th><th></th><th>Chemical Formula</th><th>Bulk Space Group (number)</th><th>Crystal System</th></tr>
<tr><td rowspan="3">Cat. 1</td><td>a</td><td>TlF</td><td rowspan="2">P4/nmm (129)</td><td rowspan="2">Tetragonal</td></tr>
<tr><td>b</td><td>MOX
(M = Lanthanide & X = Halogen):
HoOI, LaOBr, LaOCl, LaOI, LuOBr, LuOI, NdOI, YOBr

MXF
(M = Semiconductor/Metal & X = Halogen):
GeClF, PbClF, SrBrF

MHX
(M = Metal & X = I, Br):
CaHBr, CaHI, SrHBr, SrHI</td></tr>
<tr><td>c</td><td>MOX
(M = Metal & X = Halogen):
AlOCl, BiOCl, InOCl, ScOBr</td><td>Pmmn (59)</td><td>Orthorhombic</td></tr>
<tr><td colspan="2">Cat. 2</td><td>MX_2
(M = Metal & X = Halogen):
CaI_2, $CdBr_2$, $CdCl_2$, $MgBr_2$, $MgCl_2$, MgI_2, PbI_2, $ZnBr_2$, $ZnCl_2$</td><td>$P\bar{3}m1$ (164):
Mg(Br_2, Cl_2, I_2), CaI_2

$R\bar{3}m$ (166):
Cd(Br_2, Cl_2), Zn(Br_2, Cl_2)</td><td>Trigonal</td></tr>
<tr><td colspan="2">Cat. 3</td><td>MX_4
(M = Metal & X = Halogen):
PbF_4, SnF_4</td><td>I4/mmm (139)</td><td>Tetragonal</td></tr>
<tr><td colspan="2">Cat. 4</td><td>SrI_2</td><td>Pnma (62)</td><td>Orthorhombic</td></tr>
</table>

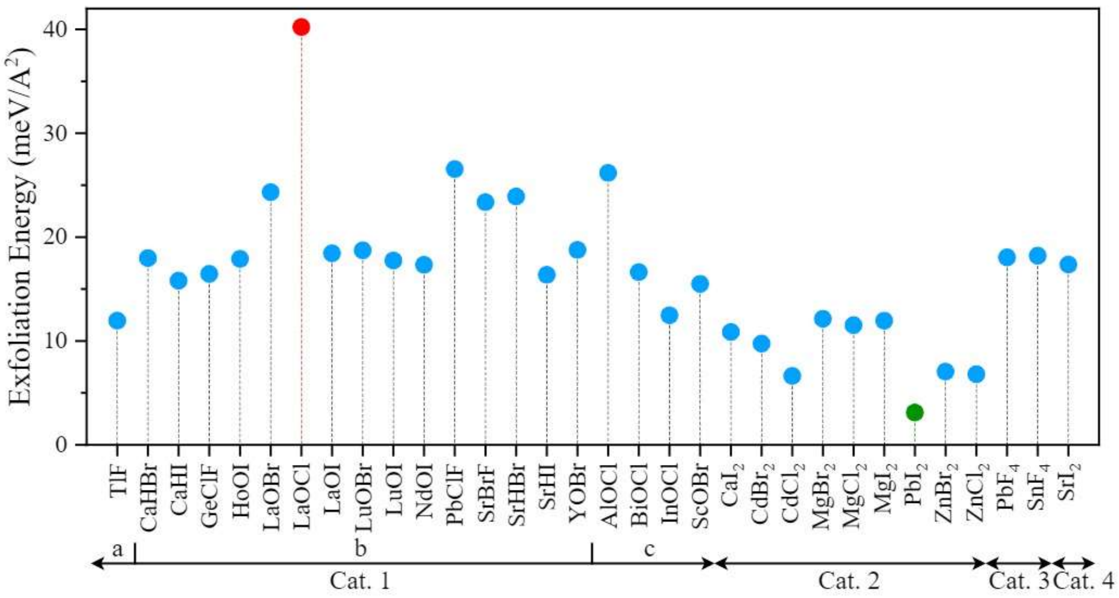

Supplementary Figure 1. The exfoliation energies (E_{ex}) obtained from PBE-DFT. The value of 100 $meV/Å^2$ is considered as the E_{ex} threshold if a material is an easily or potentially exfoliable. The green dot and the red dot respectively belong to the PbI_2 (easily exfoliable) and LaOCl (potentially exfoliable).

Supplementary Table 2. Dielectric constant values for anisotropic materials.

		Material	Bulk (ε_∞)		Bulk (ε_0)		Monolayer (ε_∞)		Monolayer (ε_0)	
			x	y	x	y	x	y	x	y
Cat. 1	c	AlOCl	3.3	2.8	10.5	5.1	3.3	2.8	10.5	5.1
		InOCl	4.1	3.6	10.3	7.6	4.0	3.5	10.2	7.5
		ScOBr	4.7	3.7	14.4	9.4	4.7	3.7	14.3	9.3

Supplementary Table 3. Our calculation for the static dielectric constants of $CdBr_2$, $CdCl_2$, and PbI_2 versus the available experimental dielectric constants [S1, S2, S3]. The frequencies at which dielectric constants are obtained are mentioned for each material. The experimental values were measured from different methods.

Experimental Data	Material	Bulk (ε_∞)	Bulk (ε_0)
		⊥	⊥
Cat. 2	$CdBr_2$	3.9 [1] (10^{14} Hz)	-
	$CdCl_2$	3.0 [2] (10^{14} Hz)	-
	PbI_2	-	6.7 [2,3] (10^{6} Hz)

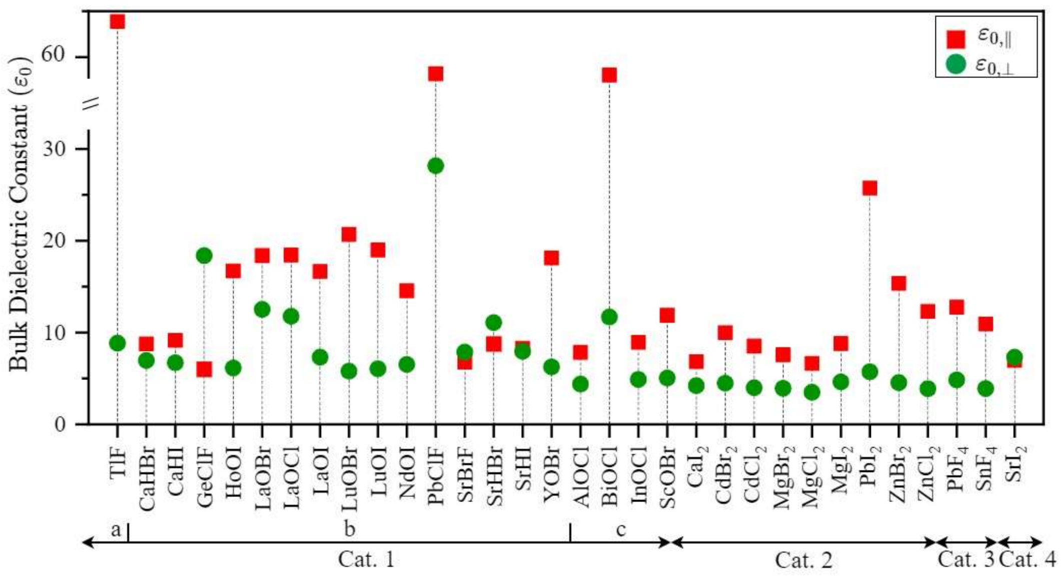

Supplementary Figure 2. The static in-plane and out-of-plane dielectric constants of 32 layered vdW materials. The in-plane dielectric values range between 6.0 (GeClF) and 63.9 (TlF), and the out-of-plane dielectric values are located in the range of 3.5 ($MgCl_2$) and 28.2 (PbClF). The red squares and green dots respectively show the in-plane and the out-of-plane static dielectric constants.

Supplementary Table 4. The PBE band gaps of bulk and monolayers, as well as HSE band gap and electron affinity of monolayers. The HSE band gap of monolayers explicitly shows that the highest and the lowest band gaps belong to AlOCl (7.38 eV) and PbI_2 (3.32 eV).

		Material	PBE Band Gap (eV)		HSE Band Gap (eV)	Electron Affinity (eV)
			Bulk	Monolayer	Monolayer	Monolayer
Cat. 1	a	TlF	3.00	3.67	4.75	1.70
	b	CaHBr	4.00	4.18	5.44	2.11
		CaHI	3.17	3.82	4.87	2.30
		GeClF	2.56	2.91	3.96	4.17
		HoOI	3.44	3.55	4.52	2.36
		LaOBr	3.68	4.05	5.68	2.37
		LaOCl	4.05	4.24	5.83	2.31
		LaOI	3.24	3.40	4.81	2.48
		LuOBr	4.28	4.48	5.79	2.35
		LuOI	3.25	3.27	4.24	2.60
		NdOI	3.62	3.74	4.72	2.46
		PbClF	3.51	3.52	4.60	3.52
		SrBrF	5.15	5.32	6.57	1.86
		SrHBr	3.72	4.29	5.42	2.01
		SrHI	3.26	3.97	5.03	2.23
		YOBr	4.42	4.68	6.00	2.15
	c	AlOCl	5.72	5.86	7.38	1.51
		BiOCl	4.00	2.75	3.74	4.54
		InOCl	2.35	2.62	4.05	4.66
		ScOBr	3.29	3.27	4.82	3.30
Cat. 2		CaI_2	3.50	3.83	4.61	2.03
		$CdBr_2$	2.73	3.25	4.47	3.63
		$CdCl_2$	3.40	3.91	5.32	3.68
		$MgBr_2$	4.30	4.79	6.01	2.21
		$MgCl_2$	5.46	6.01	7.12	2.09
		MgI_2	3.52	3.62	4.61	2.48
		PbI_2	2.25	2.58	3.32	3.67
		$ZnBr_2$	3.10	3.45	4.77	3.12
		$ZnCl_2$	4.15	4.49	6.04	2.82
Cat. 3		PbF_4	1.86	2.49	4.24	7.81
		SnF_4	3.08	3.86	6.05	6.38
Cat. 4		SrI_2	3.76	3.99	5.01	2.20

Supplementary Table 5. Monolayer band gaps from different methods. Band gap values by PBE and HSE are calculated in this work and by G_0W_0 are taken from [4]. The symbol "--" indicates that the material is not listed in [4] and the symbol "-" shows that the material is available but the G_0W_0 band gap is not reported for that material. Only the G_0W_0 data of materials in Cat. 2 with the same space group of our study are available in [4].

		Material	PBE (eV)	HSE (eV)	G_0W_0 (eV)	Experimental (eV)
Cat. 1	a	TlF	3.67	4.75	--	--
	b	CaHBr	4.18	5.44	-	-
		CaHI	3.82	4.87	--	--
		GeClF	2.91	3.96	--	--
		HoOI	3.55	4.52	--	--
		LaOBr	4.05	5.68	--	--
		LaOCl	4.24	5.83	--	--
		LaOI	3.40	4.81	--	--
		LuOBr	4.48	5.79	--	--
		LuOI	3.27	4.24	--	--
		NdOI	3.74	4.72	--	--
		PbClF	3.52	4.60	--	--
		SrBrF	5.32	6.57	--	--
		SrHBr	4.29	5.42	-	-
		SrHI	3.97	5.03	--	--
		YOBr	4.68	6.00	--	--
	c	AlOCl	5.86	7.38	-	-
		BiOCl	2.75	3.74	-	3.60 [5]
		InOCl	2.62	4.05	-	-
		ScOBr	3.27	4.82	-	-
Cat. 2		CaI_2	3.83	4.61	6.92	-
		$CdBr_2$	3.25	4.47	5.48	-
		$CdCl_2$	3.91	5.32	6.72	-
		$MgBr_2$	4.79	6.01	7.72	-
		$MgCl_2$	6.01	7.12	9.60	-
		MgI_2	3.62	4.61	5.74	-
		PbI_2	2.58	3.32	3.22	2.47 [6]
		$ZnBr_2$	3.45	4.77	5.86	-
		$ZnCl_2$	4.49	6.04	7.44	-
Cat. 3		PbF_4	2.49	4.24	-	-
		SnF_4	3.86	6.05	-	-
Cat. 4		SrI_2	3.99	5.01	--	-

Supplementary Table 6. The dielectric constant of monolayer, under four different vacuum conditions, and bilayer LaOCl.

		Vacuum (Å)	ε_∞		ε_0	
			$\perp$	$\parallel$	$\perp$	$\parallel$
LaOBr	Monolayer	15	5.32	4.66	13.21	18.24
		25	5.32	4.67	13.09	18.38
		30	5.32	4.67	13.18	18.33
		35	5.32	4.66	12.93	18.39
LaOCl	Monolayer	15	5.53	4.54	55.80	21.28
		25	5.53	4.54	67.62	22.85
		30	5.54	4.54	44.05	21.46
		35	5.53	4.54	80.68	23.73
	Bilayer	32	4.81	4.50	12.52	21.43

Supplementary Table 7. The sensitivity of dielectric constants to the one calculated from DFT for the monolayer of LaOBr and LaOCl for different vacuum sizes.

	Vacuum (Å)	$\frac{d\varepsilon_{2d}}{d\varepsilon_{SC}}$	
		$\perp$	$\parallel$
LaOBr	15	2.91×10^{2}	3.25
	25	3.88×10^{2}	3.89
	30	4.99×10^{2}	4.53
	35	5.84×10^{2}	5.17
LaOCl	15	6.02×10^{3}	3.63
	25	1.34×10^{4}	4.69
	30	7.13×10^{3}	5.45
	35	2.84×10^{4}	6.21

Supplementary Table 8. The leakage current density for *n*-MOS applications through a monolayer (1L), bilayer (2L) and three-layer (3L) of each vdW material and the Equivalent Oxide Thickness (EOT) of a monolayer. The asterisks indicate materials with a negative band-offset that are not viable as insulators for *n*-MOS applications.

n-MOS		Material	Electron Effective Mass	Leakage Current Density (A/cm^2)			EOT (nm)
				1L	2L	3L	1L
Cat. 1	a	TlF	0.521	6.48×10^{-8}	5.61×10^{-23}	7.39×10^{-30}	0.37
	b	CaHBr	1.126	3.59×10^{-13}	6.23×10^{-22}	9.82×10^{-23}	0.69
		CaHI	0.443	4.94×10^{-5}	4.32×10^{-17}	1.07×10^{-20}	0.37
		GeClF	0.542	(*)	(*)	(*)	0.27
		HoOI	1.262	9.85×10^{-13}	1.06×10^{-18}	2.62×10^{-19}	0.51
		LaOBr	1.475	4.82×10^{-11}	2.98×10^{-19}	9.83×10^{-20}	0.23
		LaOCl	1.125	7.79×10^{-8}	1.61×10^{-21}	8.73×10^{-22}	0.05
		LaOI	3.811	1.92×10^{-15}	8.17×10^{-17}	2.02×10^{-17}	0.51
		LuOBr	1.157	3.07×10^{-10}	2.67×10^{-18}	5.19×10^{-19}	0.57
		LuOI	0.656	1.66×10^{-4}	1.55×10^{-14}	3.41×10^{-15}	0.57
		NdOI	1.252	3.97×10^{-11}	4.99×10^{-17}	1.18×10^{-17}	0.55
		PbClF	0.335	2.78×10^{6}	2.92×10^{4}	5.44×10^{2}	0.19
		SrBrF	0.347	$5.22\times10\text{-}^{6}$	4.64×10^{-19}	8.68×10^{-28}	0.37
		SrHBr	0.944	1.48×10^{-11}	1.06×10^{-24}	2.66×10^{-25}	0.33
		SrHI	0.363	1.86×10^{-4}	6.24×10^{-16}	5.71×10^{-22}	0.31
		YOBr	4.663	3.82×10^{-20}	9.76×10^{-22}	1.92×10^{-22}	0.56
	c	AlOCl	1.722	5.18×10^{-29}	6.75×10^{-32}	1.03×10^{-32}	0.68
		BiOCl	1.686	(*)	(*)	(*)	0.29
		InOCl	0.412	(*)	(*)	(*)	0.66
		ScOBr	3.807	1.41	3.12×10^{-2}	5.79×10^{-3}	0.66
Cat. 2		CaI_2	3.267	6.90×10^{-15}	6.89×10^{-17}	9.09×10^{-18}	0.62
		$CdBr_2$	0.269	3.11×10^{7}	9.76×10^{5}	8.05×10^{4}	0.55
		$CdCl_2$	0.362	7.48×10^{7}	2.11×10^{6}	2.16×10^{5}	0.44
		$MgBr_2$	0.335	5.22×10^{-1}	2.61×10^{-9}	2.32×10^{-17}	0.61
		$MgCl_2$	0.443	2.59×10^{-2}	5.91×10^{-12}	3.21×10^{-21}	0.65
		MgI_2	0.988	1.12×10^{-4}	2.41×10^{-15}	3.25×10^{-16}	0.56
		PbI_2	0.250	2.32×10^{7}	1.09×10^{6}	1.03×10^{5}	0.45
		$ZnBr_2$	0.409	4.85×10^{4}	1.13×10^{1}	4.73×10^{-3}	0.54
		$ZnCl_2$	0.353	4.14×10^{3}	1.08×10^{-1}	4.66×10^{-6}	0.57
Cat. 3		PbF_4	0.690	(*)	(*)	(*)	0.18
		SnF_4	0.560	(*)	(*)	(*)	0.29
Cat. 4		SrI_2	0.878	9.33×10^{-6}	8.33×10^{-19}	1.21×10^{-21}	0.30

Supplementary Table 9. The leakage current density for *n*-MOS applications through a monolayer (1L), bilayer (2L) and three-layer (3L) of each vdW material and the Equivalent Oxide Thickness (EOT) of a monolayer. The asterisks indicate materials with a negative band-offset that are not viable as insulators for *n*-MOS applications.

p-MOS		Material	Hole Effective Mass	Leakage Current Density (A/cm^2)			EOT (nm)
				1L	2L	3L	1L
Cat. 1	a	TlF	8.234	1.69×10^{-28}	3.89×10^{-30}	7.33×10^{-31}	0.37
	b	CaHBr	0.313	9.83×10^{-19}	2.89×10^{-44}	9.73×10^{-49}	0.69
		CaHI	0.234	1.40×10^{-13}	6.15×10^{-34}	1.23×10^{-43}	0.37
		GeClF	0.678	1.46×10^{-37}	3.75×10^{-59}	1.10×10^{-59}	0.27
		HoOI	2.704	1.72×10^{-36}	7.31×10^{-38}	1.81×10^{-38}	0.51
		LaOBr	1.180	9.41×10^{-52}	2.80×10^{-58}	9.24×10^{-59}	0.23
		LaOCl	2.251	1.99×10^{-60}	5.50×10^{-61}	3.11×10^{-61}	0.05
		LaOI	1.588	2.74×10^{-43}	1.17×10^{-44}	2.88×10^{-45}	0.51
		LuOBr	0.345	4.13×10^{-28}	1.61×10^{-58}	3.13×10^{-59}	0.57
		LuOI	3.805	1.58×10^{-35}	5.58×10^{-37}	1.26×10^{-37}	0.57
		NdOI	1.878	2.15×10^{-41}	8.27×10^{-43}	1.95×10^{-43}	0.55
		PbClF	0.898	1.77×10^{-42}	1.49×10^{-59}	5.14×10^{-60}	0.19
		SrBrF	5.845	1.96×10^{-62}	8.18×10^{-64}	1.99×10^{-64}	0.37
		SrHBr	0.360	1.95×10^{-17}	9.51×10^{-42}	1.02×10^{-47}	0.33
		SrHI	0.271	7.31×10^{-16}	1.70×10^{-38}	2.58×10^{-45}	0.31
		YOBr	0.409	3.30×10^{-31}	1.19×10^{-58}	2.35×10^{-59}	0.56
	c	AlOCl	12.913	1.55×10^{-68}	2.21×10^{-70}	3.37×10^{-71}	0.68
		BiOCl	5.732	2.41×10^{-60}	1.36×10^{-61}	3.79×10^{-62}	0.29
		InOCl	4.862	5.47×10^{-66}	9.75×10^{-68}	1.64×10^{-68}	0.66
		ScOBr	1.088	2.38×10^{-56}	4.21×10^{-58}	7.81×10^{-59}	0.66
Cat. 2		CaI_2	0.300	1.28×10^{-13}	3.55×10^{-34}	3.25×10^{-46}	0.62
		$CdBr_2$	2.422	1.56×10^{-54}	1.33×10^{-56}	1.62×10^{-57}	0.55
		$CdCl_2$	2.605	5.46×10^{-70}	5.52×10^{-72}	7.20×10^{-73}	0.44
		$MgBr_2$	0.321	2.34×10^{-19}	1.18×10^{-45}	2.16×10^{-59}	0.61
		$MgCl_2$	0.451	1.23×10^{-32}	3.43×10^{-72}	1.65×10^{-75}	0.65
		MgI_2	0.238	1.14×10^{-8}	2.45×10^{-24}	1.00×10^{-39}	0.56
		PbI_2	1.294	1.26×10^{-27}	6.64×10^{-39}	1.19×10^{-39}	0.45
		$ZnBr_2$	2.901	4.14×10^{-51}	3.56×10^{-53}	4.33×10^{-54}	0.54
		$ZnCl_2$	6.431	1.24×10^{-66}	6.51×10^{-69}	6.36×10^{-70}	0.57
Cat. 3		PbF_4	1.813	(*)	(*)	(*)	0.18
		SnF_4	1.436	(*)	(*)	(*)	0.29
Cat. 4		SrI_2	1.054	3.35×10^{-24}	3.84×10^{-43}	8.00×10^{-44}	0.30

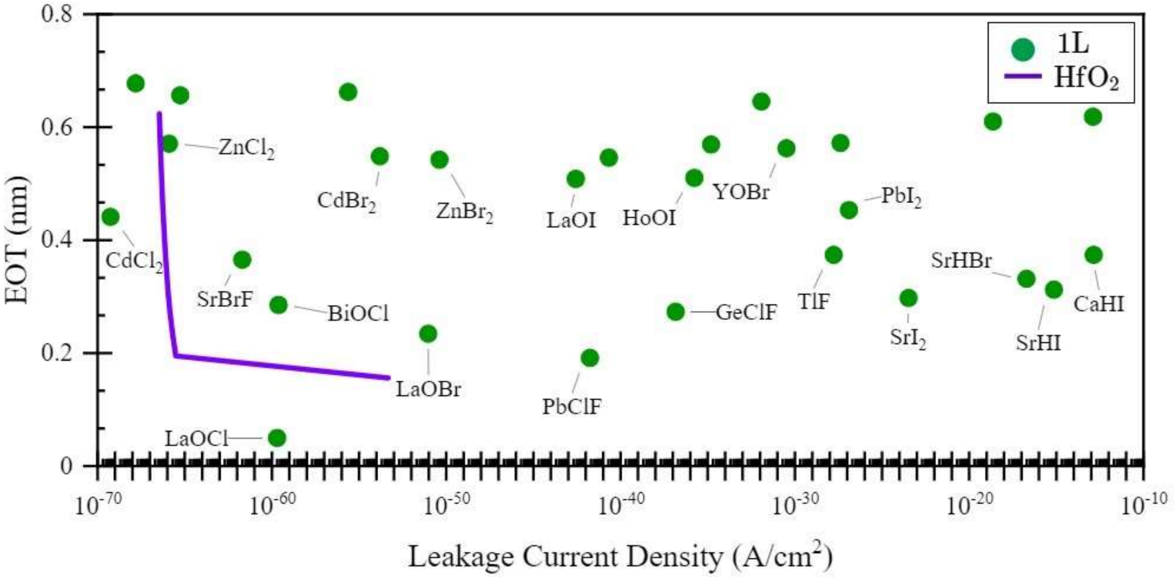

Supplementary Figure 3. EOT and leakage current density for *p*-MOS devices.

Supplementary Table 10. The lattice constants of two-dimensional (2D) monolayer structures (a_{2d}), bulk interlayer distance (d_i), Monolayer thickness (t), cutoff energies, and Monolayer supercell thickness. Δ represents the percentage difference between the bulk interlayer distance and the monolayer thickness.

		Material	Bulk	Monolayer			Cutoff Energy (eV)	Monolayer Supercell Thickness (Å)
			d_i (Å)	a (Å)	t (Å)	Δ (%)		
Cat. 1	a	TlF	6.25	3.81	6.64	6.20	560	22.55
	b	CaHBr	8.06	3.83	8.14	0.91	700	25.63
		CaHI	8.79	4.01	8.83	0.47	520	26.50
		GeClF	7.56	3.76	7.70	1.82	560	25.95
		HoOI	9.22	3.90	9.26	0.41	520	25.99
		LaOBr	7.49	4.13	7.93	5.78	560	25.80
		LaOCl	6.91	4.10	7.08	2.52	560	25.72
		LaOI	9.19	4.14	9.25	0.65	520	26.51
		LuOBr	8.34	3.74	8.38	0.49	560	25.86
		LuOI	9.20	3.84	9.23	0.35	560	25.58
		NdOI	9.26	4.07	9.29	0.33	520	25.88
		PbClF	7.26	4.13	7.44	2.52	560	26.94
		SrBrF	7.42	4.22	7.75	4.39	560	25.47
		SrHBr	7.30	4.21	7.59	3.94	700	25.38
		SrHI	8.67	4.28	8.77	1.24	520	26.07
		YOBr	8.31	3.83	8.38	0.80	560	26.36
	c	AlOCl	7.89	3.16	7.94	0.52	520	25.71
		BiOCl	7.49	3.91	7.59	1.30	520	26.30
		InOCl	8.14	3.56	8.24	1.22	560	25.95
		ScOBr	8.72	3.56	8.76	0.45	560	26.20
Cat. 2		CaI_2	7.02	4.48	7.04	0.30	450	23.70
		$CdBr_2$	6.74	6.75	6.37	-5.54	450	23.10
		$CdCl_2$	6.25	6.26	5.92	-5.35	400	24.85
		$MgBr_2$	6.30	3.84	6.34	0.59	560	23.23
		$MgCl_2$	5.86	3.64	5.90	0.67	560	22.66
		MgI_2	6.92	4.16	6.94	0.31	560	23.20
		PbI_2	7.20	14.41	7.08	-1.68	450	24.37
		$ZnBr_2$	6.35	6.63	6.34	-1.18	360	22.94
		$ZnCl_2$	5.94	6.21	5.89	-0.85	360	23.05
Cat. 3		PbF_4	4.03	5.07	4.10	1.70	520	23.30
		SnF_4	4.52	4.97	4.11	-9.02	520	23.93
Cat. 4		SrI_2	6.18	4.92	6.31	2.16	700	23.61

Supplementary Table 11. Monolayer phonon energy.

		Material	Phonon Energy (meV)					
Cat. 1	a	TlF	37.184	21.615	10.891	10.417	10.417	3.283
			3.283	3.110	3.110	0.047i	1.742i	1.742i
	b	CaHBr	136.223	120.807	120.807	111.321	101.459	101.459
			28.716	21.803	21.803	21.403	16.524	16.524
			10.265	8.354	8.354	0.032i	0.148i	0.148i
		CaHI	125.248	111.753	111.753	103.522	95.640	95.640
			25.591	21.442	21.442	19.147	14.493	14.493
			7.579	6.357	6.357	0.078i	0.150i	0.150i
		GeClF	52.846	35.470	35.470	26.415	23.755	22.562
			18.704	15.668	15.668	7.962	7.962	0.127i
			0.239i	0.239i	15.100i	15.100i	17.462i	17.462i
		HoOI	66.998	63.339	63.339	45.811	43.713	43.713
			22.039	14.684	14.273	14.273	10.590	6.797
			6.797	5.461	5.461	0.081i	0.120i	0.120i
		LaOBr	64.931	53.505	53.505	36.605	36.603	36.603
			24.468	15.642	14.447	14.447	11.950	9.738
			9.738	7.927	7.927	0.039i	0.039i	0.148i
		LaOCl	67.167	55.250	55.250	38.569	37.218	37.218
			26.176	21.130	16.993	14.359	14.359	12.495
			12.495	11.308	11.308	0.063i	0.063i	0.158i
		LaOI	62.208	50.767	50.767	36.225	35.526	35.526
			23.194	14.693	14.693	13.527	9.324	8.575
			8.575	6.255	6.255	0.077i	0.085i	0.085i
		LuOBr	71.604	70.898	70.898	47.745	47.662	47.662
			23.366	17.746	14.004	13.948	13.948	6.730
			6.730	6.178	6.178	0.074i	0.208i	0.208i
		LuOI	67.700	66.755	66.755	46.404	45.579	45.579
			21.540	14.901	14.080	14.080	10.926	6.139
			6.139	4.928	4.928	0.023i	0.137i	0.137i
		NdOI	64.160	57.739	57.739	42.618	41.534	41.534
			22.925	14.932	14.932	13.969	9.814	8.041
			8.041	6.040	6.040	0.005i	0.005i	0.019i
		PbClF	42.284	31.691	31.176	25.327	20.290	19.757
			18.174	17.295	11.347	10.075	10.031	7.302
			7.183	4.899	4.798	0.103i	0.159i	0.160i
		SrBrF	43.922	36.333	36.333	26.748	26.748	26.157
			21.500	15.307	14.475	14.475	11.999	11.999
			10.250	7.706	7.706	0.058i	0.058i	0.321i

Supplementary Table 11 (Cont.). Monolayer phonon energy

		Material	Phonon Energy (meV)					
Cat. 1	b	SrHBr	128.354	111.651	111.651	104.106	95.142	95.142
			19.030	15.379	15.379	15.061	13.406	13.406
			8.554	7.517	7.517	0.037i	0.060i	0.060i
		SrHI	118.929	105.476	105.476	96.356	91.319	91.319
			17.290	15.123	15.123	13.364	11.718	11.718
			6.826	6.268	6.268	0.169i	0.285i	0.285i
		YOBr	71.445	66.003	66.003	46.034	46.034	45.070
			31.152	19.010	18.786	18.786	13.546	8.396
			8.396	6.777	6.777	0.085i	0.191i	0.191i
	c	AlOCl	97.371	85.254	75.685	64.883	61.603	52.319
			48.004	47.361	41.601	38.837	28.807	28.492
			20.783	18.991	15.339	0.081i	0.464i	0.984i
		BiOCl	65.118	56.219	56.219	48.391	36.906	36.906
			22.798	20.845	16.454	12.025	12.025	9.134
			9.134	8.267	8.267	0.018i	0.018i	0.066i
		InOCl	74.916	57.555	55.957	50.397	42.046	35.340
			34.971	34.450	20.562	19.544	18.653	18.272
			11.070	10.031	8.501	0.067i	0.081i	0.103i
		ScOBr	69.550	69.241	57.517	50.548	45.281	41.815
			40.220	31.009	30.046	27.298	17.543	16.152
			10.541	7.455	5.181	0.095i	0.123i	0.317i
Cat. 2		CaI_2	7.007	18.585	18.585	10.904	8.174	8.174
			0.059i	0.059i	0.074i			
		$CdBr_2$	21.697	17.015	12.883	12.883	9.308	9.308
			0.056i	0.258i	0.258i			
		$CdCl_2$	27.964	25.634	17.773	17.773	14.323	14.323
			0.277i	0.277i	0.350i			
		$MgBr_2$	40.403	23.669	23.669	17.853	10.971	10.971
			0.020i	0.236i	0.236i			
		$MgCl_2$	48.444	29.664	29.664	28.266	18.372	18.372
			0.133i	0.133i	0.613i			
		MgI_2	6.130	21.542	21.542	12.659	7.388	7.388
			0.265i	0.265i	0.377i			
		PbI_2	14.775	10.683	8.765	8.765	7.338	7.338
			0.012i	0.187i	0.187i			
		$ZnBr_2$	25.920	18.806	12.815	12.815	8.891	8.891
			0.042i	0.042i	0.254i			
		$ZnCl_2$	32.856	29.705	16.498	16.498	14.846	14.846
			0.316i	0.377i	0.377i			

Supplementary Table 11 (Cont.). Monolayer phonon energy

	Material	Phonon Energy (meV)					
Cat. 3	PbF_4	74.577	64.961	51.758	51.758	22.468	17.563
		15.191	15.191	13.252	13.252	7.978	7.978
		0.450i	0.613i	0.613i			
	SnF_4	85.482	73.252	60.680	60.680	29.092	21.585
		19.426	19.426	17.998	17.998	14.703	14.703
		0.333i	0.407i	0.407i			
Cat. 4	SrI_2	17.764	17.320	14.068	13.459	13.312	13.038
		11.497	9.607	8.106	7.465	7.210	6.518
		6.264	5.329	4.156	0.015i	0.020i	0.045i

Supplementary Table 12. Bulk phonon energy.

		Material	Phonon Energy (meV)					
Cat. 1	a	TlF	25.406	22.743	12.987	12.987	10.422	6.806
			6.806	3.795	3.795	0.028i	0.059i	0.059i
	b	CaHBr	126.479	119.488	119.488	111.659	100.922	100.922
			27.806	22.145	22.145	17.092	17.085	17.085
			11.838	9.660	9.660	0.038i	0.086i	0.086i
		CaHI	117.467	112.103	112.103	104.575	96.510	96.510
			25.340	21.617	21.617	16.850	14.901	14.901
			8.910	7.105	7.105	0.075i	0.075i	0.107i
		GeClF	37.384	34.390	34.390	26.048	23.769	17.988
			16.165	16.165	13.586	12.096	12.096	0.030
			0.030	0.375	14.759	14.759i	17.788i	17.788i
		HoOI	63.421	63.421	60.774	45.085	43.809	43.809
			22.040	14.357	14.357	13.704	11.659	7.118
			7.118	6.472	6.472	0.027i	0.112i	0.112i
		LaOBr	3.627	49.716	49.716	37.492	34.503	34.503
			22.800	15.330	15.330	15.097	12.797	12.797
			12.404	10.813	10.813	0.108i	0.115i	0.115i
		LaOCl	56.313	51.679	51.679	38.484	36.285	36.285
			25.594	22.358	20.738	20.738	18.550	14.231
			14.231	13.671	13.671	0.044i	0.073i	0.073i
		LaOI	55.555	50.445	50.445	36.780	35.304	35.304
			22.349	14.031	14.031	11.834	10.539	8.524
			8.524	7.350	7.350	0.430i	0.618i	0.618i
		LuOBr	70.767	70.767	65.648	47.986	47.630	47.630
			23.321	15.942	15.498	13.982	13.982	8.223
			8.223	7.236	7.236	0.042i	0.150i	0.150i
		LuOI	66.640	66.640	61.599	45.960	45.470	45.470
			21.546	14.133	14.133	13.959	11.887	6.368
			6.368	5.940	5.940	0.045i	0.061i	0.061i
		NdOI	57.885	57.589	57.589	42.102	41.467	41.467
			22.816	15.040	15.040	12.825	10.973	8.383
			8.383	7.066	7.066	0.005i	0.005i	0.062i
		PbClF	34.549	30.071	30.071	25.835	20.862	20.862
			19.645	15.937	15.937	13.073	10.268	8.146
			8.146	5.133	5.133	0.080i	0.196i	0.196i
		SrBrF	34.755	33.956	33.956	27.131	25.792	25.792
			19.510	15.294	15.294	13.430	12.486	12.486
			11.910	10.930	10.930	0.030i	0.148i	0.148i

Supplementary Table 12 (Cont.). Bulk phonon energy

		Material	Phonon Energy (meV)					
Cat. 1	b	SrHBr	34.755	33.956	33.956	27.131	25.792	25.792
			19.510	15.294	15.294	13.430	12.486	12.486
			11.910	10.930	10.930	0.030i	0.148i	0.148i
		SrHI	109.322	103.040	103.040	96.928	90.318	90.318
			16.523	14.966	14.966	11.895	11.895	10.493
			7.775	7.180	7.180	0.037i	0.045i	0.045i
		YOBr	65.786	65.786	64.196	45.978	45.978	44.655
			30.839	18.869	18.869	16.818	15.295	8.958
			8.958	8.822	8.822	0.017i	0.017i	0.033i
	c	AlOCl	97.501	75.864	73.341	65.030	61.340	52.142
			47.470	46.963	41.514	39.079	29.643	27.497
			20.116	17.192	14.862	0.029i	0.113i	0.128i
		BiOCl	58.742	55.088	55.088	48.415	36.352	36.352
			23.856	17.628	16.733	16.733	15.916	10.178
			10.178	8.618	8.618	0.033i	0.066i	0.066i
		InOCl	74.966	56.218	52.757	50.157	42.175	35.980
			34.252	32.606	21.800	20.048	19.034	18.716
			11.899	9.983	9.823	0.010i	0.066i	0.132i
		ScOBr	69.336	62.790	57.388	50.843	45.299	41.338
			40.095	30.167	30.078	27.460	17.830	17.009
			11.154	7.106	5.803	0.019i	0.098i	0.205i
Cat. 2		CaI_2	25.456	19.181	19.181	11.636	8.527	8.527
			0.059i	0.059i	0.093i			
		$CdBr_2$	19.540	17.627	13.151	13.151	9.193	9.193
			0.005i	0.005i	0.097i			
		$CdCl_2$	27.482	24.663	18.172	18.172	15.404	15.404
			0.012i	0.012i	0.024i			
		$MgBr_2$	36.791	24.600	24.600	18.395	11.412	11.412
			0.090i	0.147i	0.147i			
		$MgCl_2$	42.276	29.736	29.736	29.726	19.227	19.227
			0.167i	0.246i	0.246i			
		MgI_2	33.844	21.999	21.999	13.362	7.873	7.873
			0.256i	0.256i	0.441i			
		PbI_2	14.674	13.418	11.257	10.648	9.218	9.218
			8.977	8.977	7.283	7.283	6.999	6.999
			3.568i	1.523i	1.523i	0.082	0.082	0.148
		$ZnBr_2$	23.560	19.551	12.444	12.444	8.657	8.657
			0.143i	0.143i	0.238i			
		$ZnCl_2$	30.867	29.940	16.837	16.837	14.611	14.611
			0.003i	0.003i	0.249i			

Supplementary Table 12 (Cont.). Bulk phonon energy

	Material	Phonon Energy (meV)					
Cat. 3	PbF_4	70.504	62.399	54.344	54.344	20.155	20.155
		17.982	15.981	15.191	15.191	13.339	13.339
		0.183i	0.242i	0.242i			
	SnF_4	79.955	70.502	62.426	62.426	25.629	25.629
		25.347	21.762	19.360	19.360	16.471	16.471
		0.021i	0.193i	0.193i			
Cat. 4	SrI_2	16.479	16.047	15.659	14.085	13.961	13.251
		13.171	13.096	12.989	12.936	12.810	12.570
		11.335	11.305	10.219	10.129	10.034	9.012
		8.638	8.541	8.469	8.020	7.453	7.325
		7.302	6.729	6.432	5.481	5.365	4.733
		4.662	4.077	3.534	0.043i	0.054i	0.069i

Supplementary References

1. R. D. Bringans and W. Y. Liang, "The dielectric functions of CdI2, CdBr2 and CdCl2," *Physica B+ C,* vol. 99, pp. 276-280, 1980.

2. D. P. Yadav, K. V. Rao and H. N. Acharya, "Dielectric properties of PbI2 single crystals.," *physica status solidi (a),* vol. 60.1, pp. 273-276, 1980.

3. S. Ekhard, B. Palosz and B. Wruck., "In situ observation of the polytypic phase transition 2H-12R in PbI2: investigations of the thermodynamic structural and dielectric properties," *Journal of Physics C: Solid State Physics,* vol. 20.26, p. 4077, 1987.

4. S. Haastrup and et al, "The Computational 2D Materials Database: high-throughput modeling and discovery of atomically thin crystals," *2D Materials,* vol. 5.4, p. 042002, 2018.

5. J. Lu and et al, "Electronic structures and lattice dynamics of layered BiOCl single crystals," *The journal of physical chemistry letters,* vol. 11.3, pp. 1038-1044, 2020.

6. M. Zhong and et al, "Large-scale 2D PbI 2 monolayers: experimental realization and their indirect band-gap related properties," *Nanoscale,* vol. 9.11, pp. 3736-3741, 2017.
